# Curing Colds

**Published:** 1874-10

**Authors:** 


### CURING COLDS.

It is gravely recommended in a medical publication as a means of breaking up a cold, to give sesqui carbonate of ammonia and morphia, that is hartshorn and opium, every three hours, and to cover up in bed with extra blankets to promote perspiration. This method is spoken of as a "new remedy" by Dr. Dobdell. But the virtue of the whole treatment is in the sweating, as our great grandmother's knew a century or two ago. Any cold can "be broken up by a good sweat," if taken within forty-eight hours of its reception, and nothing is eaten during that time but cold bread and butter. Nothing will break up a cold if neglected forty-eight hours, for then it will, like measles, take its own course of ten days or two weeks, when it "breaks" of itself and gets well, if not renewed. All the vaunted remedies for coughs and colds obtained their reputation from having been taken at the time of nature's breaking them up.

